# Regulation of 2,4-D Isooctyl Ester on *Triticum aestivum* and *Aegilops tauschii* Tillering and Endogenous Phytohormonal Responses

**DOI:** 10.3389/fpls.2021.642701

**Published:** 2021-04-28

**Authors:** Haiyan Yu, Hailan Cui, Jingchao Chen, Pingping Chen, Meijing Ji, Songtao Huang, Xiangju Li

**Affiliations:** Key Laboratory of Weed Biology and Management, Institute of Plant Protection, Chinese Academy of Agricultural Sciences, Beijing, China

**Keywords:** *Triticum aestivum*, *Aegilops tauschii*, Tiller bud, RNA-Seq, phytohormones, 2, 4-D isooctyl ester

## Abstract

Tillering is an important agronomic trait essential for the yield of *Triticum aestivum* and the propagation of *Aegilops tauschii*. However, the effect of phytohormones on *T. aestivum* and *Ae. tauschii* tillering and the underlying regulatory mechanisms remain poorly understood. In the study, we found that *T. aestivum* and *Ae. tauschii* exhibited different tillering sensitivities to the auxin herbicide 2,4-D isooctyl ester. At 3 days post-application, tiller bud growth was inhibited by 77.50% in *T. aestivum*, corresponding to 2.0-fold greater inhibition than that in *Ae. tauschii* (38.71%). Transcriptome analysis showed that differentially expressed genes (DEGs) in the *T. aestivum* response to 2,4-D isooctyl ester were mainly enriched in plant hormone metabolism and signal transduction pathways, but similar changes were not observed in *Ae. tauschii*. Among that, the auxin biosynthesis and signaling induced by 2,4-D isooctyl ester was quite different between the two species. A total of nine candidate genes involved in varied tillering responses were selected from the DEGs and validated by quantitative real-time PCR. Endogenous hormone levels were assayed to further verify the RNA-seq results. After 2,4-D isooctyl ester treatment, a significant increase in abscisic acid (ABA) levels was observed in *T. aestivum*, whereas ABA levels were relatively stable in *Ae. tauschii*. The herbicide induced more cytokinin (CTK) accumulation in *Ae. tauschii* than in *T. aestivum*. External ABA clearly restricted tiller bud growth in both *T. aestivum* and *Ae. tauschii*, while 6-benzyl aminopurine had no significant effect. These results indicate that ABA and CTK may be related with 2,4-D isooctyl ester-regulated tillering differences between the two species, which will help to further understand the mechanism of the auxin-mediated regulation of tillering

## Introduction

Wheat (*Triticum aestivum* L), one of the most important cereal crops worldwide, is severely infested by the notorious grass weed *Aegilops tauschii* in China, resulting in considerable yield losses (Zhang et al., 2007; Zhao et al., 2019). However, as the D-genome progenitor of *T. aestivum*, *Ae. tauschii* shares many similar biological and genetic characteristics with *T. aestivum*, intensifying the difficulties in developing selective herbicides that can be applied to control it (Tahernezhad et al., 2010; Yu and Li, 2018). Tillering is an important agronomic trait of Poaceae that greatly determines seed output (Lecarpentier et al., 2019). Strong tillering ability is of great value for *T. aestivum* to satisfy the increasing global demand for food, whereas in *Ae. tauschii*, a strong tillering ability would accelerate its reproduction and expansion to new habitats by improving the propagation coefficient.

The occurrence of tillers is generally initiated by the establishment of tiller buds, followed by their elongation (Li et al., 2003). It is well known that the outgrowth of tiller buds can be manipulated by several phytohormones. Among them, auxin, as the earliest discovered phytohormone, has long been reported to negatively regulate tiller bud growth (Shimizu-Sato et al., 2009). However, auxin cannot enter the tiller bud, resulting in an indirect inhibitory effect on tillering (Booker et al., 2003). Currently, there are two models explaining the auxin-mediated indirect inhibition of tillering. The auxin transport canalisation hypothesis emphasizes that auxin transport from the tiller bud to the main stem is critical for tiller bud activation, and this effect can be restrained by auxin transported downwards through the xylem (Bennett et al., 2006; Prusinkiewicz et al., 2009). The other hypothesis proposes that there is a second messenger that transmits auxin signals to the tiller bud (Snow, 1937; Sachs and Thimann, 1967). As expected, external application of auxin had an inhibitory influence on the growth of tiller buds. For example, spraying indole-3-acetic acid (IAA) and naphthyl acetic acid (NAA, a synthetic auxin) on rice significantly restricted tiller bud elongation (Liu et al.,2011a,b; Xu et al., 2015). In wheat, tiller bud development was also completely inhibited by exogenous application of IAA (Cai et al., 2018). 2,4-D isooctyl ester, a derivative of 2,4-D (a synthetic auxin), is an auxin herbicide popularly used for broadleaf weed control in some Poaceae crops, such as wheat and maize (Wu et al., 2008). Our previous study indicated that although *T. aestivum* and *Ae. tauschii* are closely related genetically, they exhibit different sensitivities to 2,4-D isooctyl ester, including different effects on tillering. However, the mechanism underlying the different tillering responses of the two plant species to 2,4-D isooctyl ester is still not well understood.

Regarding the mechanism explaining external auxin-mediated regulation of tiller bud growth, Liu et al. (2011b) reported that auxin arrested the growth of tiller buds in rice by negatively regulating the expression of the rice gene adenosine phosphate isopentenyltransferase (*OsIPT*), which encodes a crucial enzyme involved in cytokinin (CTK) biosynthesis and levels in nodes. Furthermore, external auxin could also affect strigolactone (SL) synthesis, which is considered the newest phytohormone implicated in branching (Umehara et al., 2008; Xu et al., 2015). *FINE CULM 1* (*FC1*), which is homologous to maize *TEOSINTE BRANCHED1* (*TB1*) and *Arabidopsis BRANCHED 1* (*BRC1*), is strongly believed to participate in tiller bud inhibition in rice (Doebley et al., 1997; Takeda et al., 2003; Aguilar-Martínez et al., 2007). Changes in *FC1* expression patterns were markedly delayed by sprayed application of external auxin on rice, and this effect was mediated by alterations in SL and CTK downstream signals (Xu et al., 2015). In wheat, it was proposed that changes in tiller bud elongation caused by exogenous hormones were closely associated with changes in endogenous zeatin contents (Cai et al., 2018). Based on this, we hypothesized that endogenous hormone metabolism in the two plant species *T. aestivum* and *Ae. tauschii*., respond differently to external 2,4-D isooctyl ester application. Based on the above speculation, the identity of the main plant hormones involved in different tillering sensitivities needs to be clarified further.

Currently, very little is understood regarding the effect of 2,4-D isooctyl ester on tiller bud growth in *T. aestivum* and *Ae. tauschii*. Moreover, the mechanism for the different tillering responses to 2,4-D isooctyl ester in the two species has been obscure. Therefore, the objectives of this study are to (i) evaluate the effect of 2,4-D isooctyl ester on the outgrowth of tiller buds in *T. aestivum* and *Ae. tauschii*; (ii) identify metabolic pathways and candidate genes involved in the diverse tillering sensitivities to 2,4-D isooctyl ester by transcriptomic analysis and validate the identified candidates using quantitative real-time PCR (qPCR); (iii) assess the dynamics of endogenous hormone levels after 2,4-D isooctyl ester spray application; and (iv) ascertain the effect of exogenous abscisic acid (ABA) and 6-benzyl aminopurine (6-BA, a synthetic CTK) on tiller bud elongation in *T. aestivum* and *Ae. tauschii* based on the above results of endogenous hormone contents. Undoubtedly, such results will be highly valuable to further understand the regulatory mechanism of auxin on tillering growth and provide new ideas for solving the challenge of plant hormone selectivity between *T. aestivum* and *Ae. tauschii*.

## Materials and Methods

### Plant Materials and Growth Conditions

*Ae. tauschii* seeds were collected from Henan Province, China, which is the main wheat production province with wide spread of the weed species. One typical cultivar of winter wheat popularly planted in Henan Province, Xinaikang58, was used in this study. *Ae. tauschii* and *T. aestivum* seeds were sown in 9-cm-diameter pots containing a mixture of soil, vermiculite and organic fertilizer (3:1:1, v/v/v). All pots were placed in a growth chamber under a photoperiod of 14 h light and 10 h dark (18°C/13°C), with a light intensity of 500 μmol m^–2^s^–1^, and a relative humidity of 65%. Plants were thinned to maintain six healthy and uniform seedlings per pot one week after planting.

### The Effect of 2,4-D Isooctyl Ester on Tiller Bud Outgrowth

2,4-D isooctyl ester 87.5% emulsifiable concentrate (EC) was provided by Shandong Binnong Technology Co., Ltd. (Binzhou, China). Thirteen-day-old seedlings, when they reached the 2- to 3-leaf stage and the length of the tiller bud in the axil of the first leaf was less than 5 mm, were treated with 2,4-D isooctyl eater at concentrations of 525 (recommended concentration), 1050, 2100, 4200, 8400, 16800, and 33600 g a.i. ha^–1^ (a.i., active ingredient). Deionized water spray was used as a control. Spraying was performed using an ASS-3 Walking Spray Tower (450 L ha^–1^; National Engineering Research Center for Information Technology, Beijing, China). The lengths of the tiller buds located at the axil of the first leaf at 3 and 6 days after treatment (DAT) were measured in 50 seedlings from each treatment group, and the experiment was conducted with three independent replicates.

### Sample Preparation for RNA Sequencing, qPCR Validation, and Phytohormone Analysis

**FIGURE 1:**
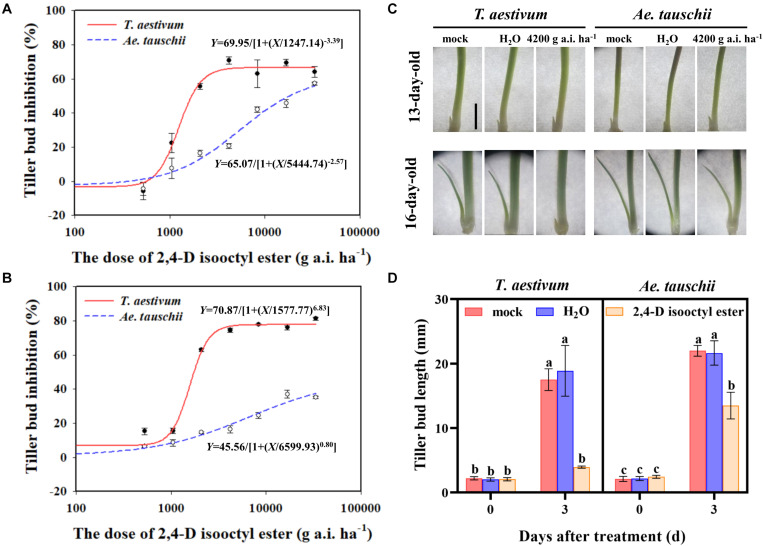
**(A,B)** Dose response of *Triticum aestivum* and *Aegilops tauschii* tiller bud growth to 2,4-D isooctyl ester at 3 **(A)** and 6 **(B)** days after treatment (DAT). **(C)** Phenotypes of *T. aestivum* and *Ae. tauschii* tiller buds in the axil of the first leaf at 0 DAT (13-day old) and 3 DAT (16-day-old). The scale bar represents 5 mm. **(D)** The length of tiller buds located at the axil of the first leaf in *T. aestivum* and *Ae. tauschii* at 0 and 3 days after treatment with 4200 g a.i. ha^– 1^ 2,4-D isooctyl ester. Error bars represent the mean ± SD (*n* = 3). Different letters represent significant differences (ANOVA, Student-Newman-Keuls test, *P* < 0.05).

**FIGURE 2:**
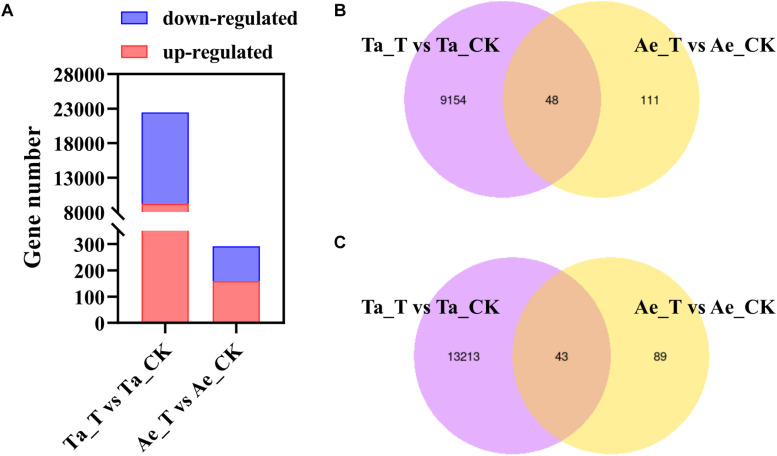
Differentially expressed genes (DEGs) in *T. aestivum* and *Ae. tauschii* response to 2,4-D isooctyl ester. **(A)** Numbers of upregulated DEGs and downregulated DEGs. **(B,C)** Venn diagram of two comparison groups of upregulated DEGs **(B)** and downregulated DEGs **(C)**. Ta_T, *T. aestivum* with 2,4-D isooctyl ester treatment; Ta-CK, *T. aestivum* without 2,4-D isooctyl ester treatment; Ae_T, *Ae. tauschii* with 2,4-D isooctyl ester treatment; Ae-CK, *Ae. tauschii* without 2,4-D isooctyl ester treatment.

As shown in Figures 1A,B, 2,4-D isooctyl ester at a concentration of 4200 g a.i. ha^–1^ was used for RNA sequencing, qPCR validation and phytohormone analysis. 2,4-D isooctyl ester was sprayed on 13-day-old seedlings as described above, and untreated plants and deionized water spray were considered as a mock and a control, respectively. The lengths of the tiller buds in the axil of the first leaf were measured at 0 DAT and 3 DAT. Moreover, at 3 DAT, tiller buds were sampled from untreated and herbicide treated plants for RNA-seq immediately after measuring their lengths. The samples from *T. aestivum* with and without 2,4-D isooctyl ester treatment were recorded as Ta_T and Ta_CK, respectively; for *Ae. tauschii* corresponding values were recorded as Ae_T and Ae_CK, respectively. One sample consisted of tiller buds from twenty plants with three independent replicates; thus, a total of twelve samples were collected. For qPCR validation, tiller buds located in the axil of the first leaf were collected at 0, 1, 3, and 5 DAT. Each treatment group contained twenty plants, and the experiment was carried out with three independent replicates. Due to the difficulty of obtaining enough tiller buds for detecting endogenous hormones, 0.5 cm of the basal part of the stem (BPS) was cut at 0, 6, 12, 24, and 72 h after treatment (HAT). Each treatment group included a bulk of BPS collected from twenty plants, and the experiment was repeated three times.

### RNA Extraction and Library Construction and Sequencing

Total RNA from twelve samples was extracted with TRIzol, and RNA concentration, quality and RIN values were assessed using an Agilent 2100 (Agilent Technologies, CA, United States). mRNA was obtained by enriching qualified RNA using magnetic beads equipped with oligo (dT). The library construction method was described by Yu et al. (2020a). The quality of the constructed libraries was strictly monitored before sequencing with an Illumina HisSeqTM 4000 (Illumina, CA, United States).

### Sequence Alignment and Differential Expression Analysis

Quality control of raw reads was performed to remove adapter sequences with Palindrome mode of Trimmomatic version0.33 software. The main parameters of ILLUMINACLIP were set as follows: seed mismatches, 2; palindrome clip threshold, 30; simple clip threshold, 10, minAdapterLength, 8; keepBothReads, false. Besides, reads containing more than 10% uncertain nucleotides and reads containing more than 50% low-quality nucleotides were also removed. Clean reads were mapped against the *T. aestivum* reference genome (*Triticum*_*aestivum*_release42) with STAR software (Dobin et al., 2013). The read length was 150 bp and the mapped parameters were set as follows: mismatch, 2; runModel alignReads, twopassModel Basic; runThread, 15; alignIntroMax, 30000; outSAMtype BAM, SortedByCoordinate; outFilterMultimapNmax, 20; outSAMstrandField, introMotif; outFilterIntroMotifs, RemoveNoncanonical; outSAMunmapped, Within. The gene expression level is described as fragments per kilobase of exon model per million mapped reads (FPKM). Total mapped reads are used for estimating gene expression levels with union model of HTSeq software. Differential expression analysis was performed by DESeq (Anders and Huber, 2010). Genes with *q* value (adjusted *p* value) < 0.05 were considered differentially expressed genes (DEGs). The comparison of gene expression differences was carried out between Ta_T and Ta_CK (Ta_T vs Ta_CK) and Ae_T and Ae_CK (Ae_T vs Ae_CK). All DEGs were subjected to Gene Ontology (GO) enrichment analysis with the GOseq R package (Young et al., 2010). Additionally, Kyoto Encyclopedia of Genes and Genomes (KEGG) pathway analysis was also performed on the DEGs.

### qPCR Validation

Total RNA was extracted as described above, and a total of 1000 ng RNA was used for synthesis of first-strand cDNA following the procedure of TransScript^®^ All in One First-Strand cDNA Synthesis SuperMix for qPCR (Transgene, Beijing, China). qPCR was carried out with Bestar^®^ SybrGreen qPCR Mastermix (DBI^®^ Bioscience, Ludwigshafen, Germany) in a 20 μL reaction volume containing 10 μL of qPCR Mastermix, 8 μL of ddH_2_O, 0.5 μL of forward and reverse primers and 1 μL cDNA using an ABI 7500 Fast Sequencer. *Tatubulin* and *Aetubulin* were regarded as reference genes to normalize the target gene expression levels in *T. aestivum* and *Ae. tauschii*, respectively. The 2^–ΔΔ*CT*^ method was used to calculate the gene expression level. All qPCR primers are shown in Supplementary Table 1.

### Endogenous Hormone Detection

The contents of ABA, IAA, and CTK, including trans-zeatin (tZ), trans-zeatin-riboside (tZR), N6-(delta 2-isopentenyl)-adenine (iP) and N6-isopentenyladenosine (iPA), together with gibberellin A3 (GA_3_) and 1-aminocyclopropanecarboxylic acid (ACC), a precursor to ethylene (ETH), in BPS were assayed following a previously published method (Šimura et al., 2018; Yu et al., 2020a). The MRM parameters of the above detected plant hormones are displayed in Supplementary Table 2.

### External Application of ABA and 6-BA

To ascertain the effect of exogenous ABA and CTK on tiller bud outgrowth, ABA and 6-BA were applied to *T. aestivum* and *Ae. tauschii*. Uniform 13-day-old seedlings, when they reached the 2- to 3-leaf stage and the length of the tiller bud in the axil of the first leaf was less than 5 mm, were treated with ABA or 6-BA at concentrations of 300, 600, and 1200 mg L^–1^. Deionized water was applied as control. Plants were sprayed twice with an ASS-3 Walking Spray Tower so the dilutions covered the leaves well. After treatment, all plants were placed under the same conditions as before. The lengths of the tiller buds located at the axil of the first leaf were assayed at 7 DAT. Each treatment included five pots containing six seedlings each, and the experiment was independently repeated three times.

### Statistical Analysis

All experiments were performed with three replicates, and the means and standard deviations were calculated. The 2,4-D isooctyl ester-dose response of tiller bud length was fit to a logistic nonlinear regression model using SigmaPlot software (v. 12.5, Systat Software, Point Richmond, CA):

*Y*=*C* + (*D*−*C*)/[1 + (*X*/*X*_0_)^*b*^] (Seefeldt et al., 1995)

where *C* and *D* are the lower and upper limits of the response, respectively, *X* is the dose or concentration of herbicide, *X*_0_ is the herbicide dose or concentration resulting in 50% growth inhibition, *b* is the slope at the half maximal effective dose (*X*_0_), and *Y* is the response at herbicide concentration or dose *X*. ANOVA was employed to evaluate the effect of external treatment with 2,4-D isooctyl ester, ABA and 6-BA on the elongation of tiller buds using IBM SPSS 21.0 software. The significance of the difference was determined by Student-Newman-Keuls (SNK) multiple comparisons at the 5% probability level. The data of relative gene expression levels and contents of endogenous hormones were subjected to t-test at the 5% probability level using SPSS to compare significant difference between mock and treatment groups as well as between *T. aestivum* and *Ae. tauschii* at the same time, respectively.

## Results

### Different Tillering Sensitivities to 2,4-D Isooctyl Ester Between *T. aestivum* and *Ae. tauschii*

External 2,4-D isooctyl ester spraying had a significant inhibitory effect on the outgrowth of tiller buds in *T. aestivum* as well as in *Ae. tauschii*. However, the restricted tiller bud growth was distinctly different between the two plant species. At 3 DAT, the 2,4-D isooctyl ester dose required for 50% growth inhibition of tiller buds was estimated at approximately 1247.14 g a.i. ha^–1^ in *T. aestivum* and approximately 5444.74 g a.i. ha^–1^ in *Ae. tauschii* (Figure 1A), indicating that *Ae. tauschii* tillering was more tolerant to 2,4-D isooctyl ester than *T. aestivum*. A similar phenomenon was also observed at 6 DAT, and the 2,4-D isooctyl ester dose resulted in 50% growth inhibition of tiller buds in *T. aestivum* and *Ae. tauschii* occurred at approximately 1577.74 and 6599.94 g a.i. ha^–1^, respectively (Figure 1B).

As shown in Figures 1A,B, the inhibitory effect on tiller buds was largely different between *T. aestivum* and *Ae. tauschii* when 4200 g a.i. ha^–1^ 2,4-D isooctyl ester was externally applied to plants. Thus, this concentration was selected for further analysis. To confirm the above phenomenon, 2,4-D isooctyl ester at a concentration of 4200 g a.i. ha^–1^ was sprayed on *T. aestivum* and *Ae. tauschii* again. Consistently, tiller bud growth inhibition by 2,4-D isooctyl ester was more obvious in *T. aestivum* than in *Ae. tauschii* (Figure 1C). In *T. aestivum*, no distinct tiller bud elongation was detected at 3 DAT compared with 0 DAT after treatment with 4200 g a.i. ha^–1^ 2,4-D isooctyl ester. At 3 DAT, 77.50% growth inhibition of tiller buds relative to untreated plants was observed in *T. aestivum*, representing 2.0-fold greater inhibition than that in *Ae. tauschii* (38.71%) (Figure 1D). Besides, in the two plant species, there was no significant difference between untreated and deionized water treated plants, thus untreated plants were used for further analysis.

### More Differentially Expressed Genes Were Detected in *T. aestivum* Than in *Ae. tauschii*

A total of 743.2 million raw reads ranging from 57.6 million reads to 71.2 million reads per library were obtained from 12 RNA libraries after RNA-seq. After strict quality control, 95.60% to 96.54% of the raw reads per library were clean reads, 96.30% to 96.84% of the clean reads were successfully mapped to the *T. aestivum* reference genome, and 88.14% to 91.49% were uniquely mapped (Table 1). For *T. aestivum*, 22458 DEGs were detected in tiller buds after 2,4-D isooctyl ester treatment, with 9202 upregulated genes and 13256 downregulated genes. A total of 291 DEGs were noted in *Ae. tauschii* tiller buds, of which 159 genes were upregulated and 132 genes were downregulated (Figure 2A). Among the upregulated genes, 48 DEGs identified in *T. aestivum* were also noted in *Ae. tauschii* (Figure 2B). *T. aestivum* and *Ae. tauschii* shared 43 downregulated DEGs (Figure 2C).

**TABLE 1:** Summary of RNA sequencing and mapping using the *Triticum aestivum* genome (*Triticum*_*aestivum*_release42) as the reference^a^.

Sample	Total reads	Clean reads	GC (%)	Total mapped (%)	Uniquely mapped (%)	Multiple mapped (%)
Ae_CK_1	59254398	56622530	56.78	96.60	88.14	8.46
Ae_CK_2	58503174	55903948	56.73	96.49	88.39	8.10
Ae_CK_3	59984390	57630792	56.61	96.63	88.61	8.02
Ae_T_1	71239568	68776962	56.53	96.74	88.93	7.82
Ae_T_2	50419070	48464608	56.83	96.74	89.16	7.58
Ae_T_3	69080274	66379044	56.91	96.54	88.68	7.86
Ta_CK_1	65435612	62931462	56.19	96.46	90.70	5.76
Ta_CK_2	65808286	63386554	55.94	96.84	91.28	5.56
Ta_CK_3	64779606	62228122	56.99	96.84	91.49	5.35
Ta_T_1	57587418	55056354	56.02	96.56	90.83	5.74
Ta_T_2	61551270	59368730	55.93	96.56	91.30	5.25
Ta_T_3	59528134	57077778	55.73	96.30	90.50	5.81

*^*a*^Columns represent the number of raw sequencing reads, the number of clean reads, GC content and ratio of sequences mapped to the genome. Numerical values 1, 2, and 3 indicate the different biological replicates. Ae_CK, *Aegilops tauschii* without 2,4-D isooctyl ester treatment; Ae_T, *Aegilops tauschii* 72 h after 2,4-D isooctyl ester treatment; Ta_CK, *Triticum aestivum* without 2,4-D isooctyl ester spray; Ta_T, *Triticum aestivum* 72 h after 2,4-D isooctyl ester treatment.*

### GO and KEGG Enrichment Analysis

GO enrichment analysis was conducted to assess the specific functions of all DEGs. In *Ae. tauschii*, “lipid metabolic process” and “carbohydrate metabolic process” were the top enriched GO terms in the “biological process” group. “Catalytic activity” and “oxidoreductase activity” were significantly enriched in the “molecular function” group. In *T. aestivum*, “small molecule metabolic process” and “organic acid metabolic process” were the most enriched GO terms in the “biological process” group. “Plastid stroma,” “chloroplast stroma,” “chloroplast ribulose bisphosphate carboxylase complex,” and “ribulose bisphosphate carboxylase complex” were significantly enriched in the “cellular component” group. “Oxidoreductase activity” was the top category in the “molecular function” group (Supplementary Figure 1).

**FIGURE 3:**
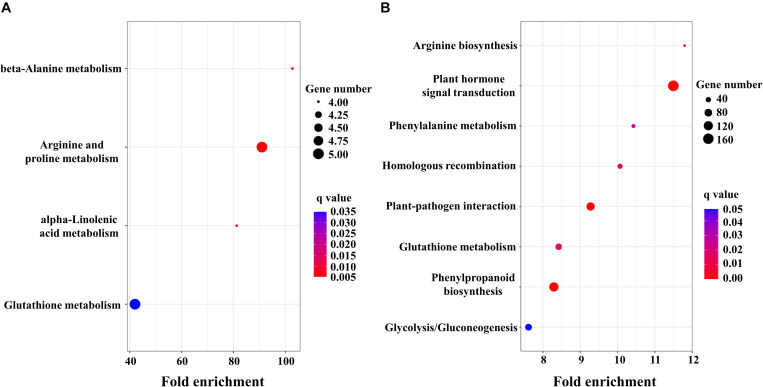
KEGG enrichment analysis of DEGs in *Ae. tauschii* **(A)** and *T. aestivum* **(B)**. Fold enrichment in a pathway means the ratio of the number of observed DEGs and expected DEGs.

To obtain detailed insight into the functional enrichment classification of the upregulated DEGs, KEGG analysis was carried out. *T. aestivum* displayed more significantly enriched pathways (*q* < 0.05) than *Ae. tauschii*, including arginine biosynthesis, plant hormone signal transduction, phenylalanine metabolism, homologous recombination, plant-pathogen interaction, glutathione metabolism, phenylpropanoid biosynthesis and glycolysis/gluconeogenesis (Figure 3B). In contrast, beta-alanine metabolism, arginine and proline metabolism, alpha-linolenic acid metabolism and glutathione metabolism were the predominantly enriched pathways (*q* < 0.05) in *Ae. tauschii* (Figure 3A). Glutathione metabolism was the only shared pathway that was highly enriched in both *T. aestivum* and *Ae. tauschii*, which is most likely related to 2,4-D isooctyl ester metabolism. The remaining significantly enriched pathways were uniquely induced by 2,4-D isooctyl ester in *T. aestivum* or *Ae. tauschii*. Among them, of particular note was the plant hormone signal transduction pathway, which was the most significantly induced responsive pathway in *T. aestivum* and might be responsible for the differences in tillering responses to 2,4-D isooctyl ester between the two plant species (Figure 3).

### Differentially Expressed Genes Analysis

**FIGURE 4:**
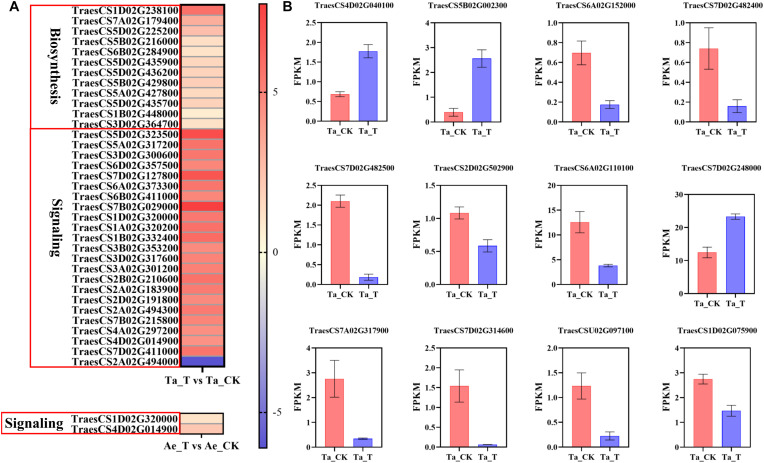
**(A)** The expression level of DEGs related with auxin biosynthesis and signal transduction in *T. aestivum* and *Ae. tauschii* based on log_2_ (fold change) with untreated plants as control. DEGs related with auxin signal transduction in *T. aestivum* with | log_2_ (fold change)| over 4 are shown. Each row represents one gene. **(B)** The fragments per kilobase of exon model per million mapped reads (FPKM) values of DEGs associated with tiller bud development in *T. aestivum*. Error bars represent the mean ± SD (*n* = 3). Other abbreviations were as shown in Figure 2.

Given the above speculation, plant hormone metabolism and signal transduction pathways were further analyzed. Compared with *Ae. tauschii*, many more DEGs were highly enriched in the plant hormone synthesis and signal transduction pathways in *T. aestivum* (Supplementary Figure 2). For example, a total of 13 DEGs related to auxin biosynthesis were identified in *T. aestivum*, but none in *Ae. tauschii*. Additionally, 155 DEGs were significantly enriched in the auxin signal transduction pathway in *T. aestivum*, while only two DEGs were noted in *Ae. tauschii* (Supplementary Figures 2A,B). Similar results were also found in the CTK, ABA, gibberellin (GA), and ETH synthesis and signal transduction pathways (Supplementary Figures 2C–J). Therefore, it was speculated that the differential impact of 2,4-D isooctyl ester on tillering growth in the two plant species was attributable to different plant hormone metabolism and signal transduction responses. Furtherly, increased expression levels of DEGs involved in auxin biosynthesis were observed in tiller buds of *T. aestivum* with 2,4-D isooctyl ester treatment, which may result in higher accumulation of IAA in tiller buds. Among them, *TAA* (TraesCS1D02G238100) differentially expressed more than 5-fold in tiller buds of treated seedlings compared with untreated seedlings. During auxin signal transduction in *T. aestivum*, except for transport inhibitor response (*TIR1*) protein, other genes (*AUX1*, *AUX/IAA*, *ARF*, *SAUR*, and *GH3*) exhibited different expression levels between untreated and treated groups. Among these DEGs, most of them exhibited markedly higher expression levels. Besides, the expression levels of *GH3* (TraesCS1D02G320000) and *SAUR* (TraesCS4D02G014900) were highly enhanced in both *T. aestivum* and *Ae. tauschii* after 2,4-D isooctyl ester treatment, whereas the increasing degree of expression was greater in *T. aestivum* (Figure 4A). Clearly, auxin biosynthesis and signaling induced by 2,4-D isooctyl ester was quite different between the two plant species, suggesting that auxin may participate in the regulation of tillering through influencing the balances of endogenous auxin metabolism and signaling.

Furthermore, some genes associated with tiller bud growth were differentially expressed after 2,4-D isooctyl ester spray in *T. aestivum*, consistent with its largely restricted tiller bud growth. *TB1* was reported to negatively regulate the tiller bud outgrowth (Wang et al., 2018). In *T. aestivum*, *TB1* (TraesCS4D02G040100 and TraesCS5B02G002300) exhibited higher expression level in 2,4-D isooctyl ester treated plants than in untreated plants (Figure 4B). It is well believed that MIR156-SPL (*SQUAMOSA PROMOTER BINDING PRTEIN-LIKE*) module participates in the regulation of plant architecture, including tillering (Wang and Wang, 2015). In treated *T. aestivum* plants, the expression levels of several genes belonging to *SPL* gene family (TraesCS6A02G152000, TraesCS7D02G482400, TraesCS7D02G482500, TraesCS2D02G502900, TraesCS6A02G110100, and TraesCS7D02G248000) differed from that in untreated plants (Figure 4B). Besides, Zhao et al. (2019) found that *DWARF27*-B (*D27*-B) controls tiller bud development in hexaploidy wheat. In present study, a total of four DEGs belonging to *D27* gene family were identified from Ta_T vs Ta_CK (TraesCS7A02G317900, TraesCS7D02G314600, TraesCSU02G097100, and TraesCS1D02G075900) and had weaker expression level in treated plants compared with untreated plants (Figure 4B). Collectively, these DEGs may result in restricted tiller bud outgrowth of *T. aestivum* seedlings with 2,4 isooctyl ester treatment.

### Candidate Gene Selection and qPCR Validation

Based on the GO function and KEGG analysis, DEGs implicated in plant hormone synthesis and signal transduction were considered candidate genes that might be related to differential tillering responses to 2,4-D isooctyl ester spray between *T. aestivum* and *Ae. tauschii*. Moreover, these candidate genes were expressed in both *T. aestivum* and *Ae. tauschii* with an absolute value of log_2_| fold change (Ta_T vs Ta_CK)| over 4 or an absolute value of log_2_| fold change (Ae_T vs Ae_CK)| over 1. Thus, a total of nine candidate genes were selected from the transcriptome analysis (Table 2). Two genes (TraesCS1D02G320000 and TraesCS2D02G191800) belong to the *auxin-responsive protein* (*GH3*) family. Two genes (TraesCS1D02G237200 and TraesCS1D02G157000) were annotated to the *cytokinin dehydrogenase* (*CKX*) family. One gene (TraesCS6D02G067900) encoding *histidine-containing phosphor transfer protein 1-like* (*AHP1*) and one gene (TraesCS3D02G124500) annotated to the *gibberellin 3-beta-dioxygenase 2* (*GA_3_ox2*) family were identified. Additionally, one gene (TraesCS5D02G383500) belonging to the *9-cis-epoxycarotenoid dioxygenase* (*NCED*) family, one gene (TraesCS3D02G238000) annotated to the *protein phosphatase 2C* (*PP2C*) family and one gene (TraesCS7D02G516200) encoding *1-aminocyclopropane-1-carboxylate oxidase* (*ACO*) were obtained.

**TABLE 2:** Candidate genes involved in different tillering responses to 2,4-D isooctyl ester of *Triticum aestivum* versus *Aegilops tauschii* selected from transcriptome analysis.

Family	Gene ID	Log_2_[fold change (Ta_T vs Ta_CK)]	Description
GH3	TraesCS1D02G320000	5.469	Auxin-responsive protein
	TraesCS2D02G191800	4.719	Auxin-responsive protein
CKX	TraesCS1D02G237200	−4.276	Cytokinin dehydrogenase 9
	TraesCS1D02G157000	0.983	Cytokinin dehydrogenase 6
AHP	TraesCS6D02G067900	−4.299	Histidine-containing phosphor transfer protein 1-like
GA_3_ox2	TraesCS3D02G124500	−5.644	Gibberellin 3-beta-dioxygenase 2-2
NCED	TraesCS5D02G383500	4.063	9-cis-epoxycarotenoid dioxygenase
PP2C	TraesCS3D02G238000	4.334	Protein phosphatase 2C 8
ACO	TraesCS7D02G516200	4.374	1-Aminocyclopropane-1-carboxylate oxidase

**FIGURE 5:**
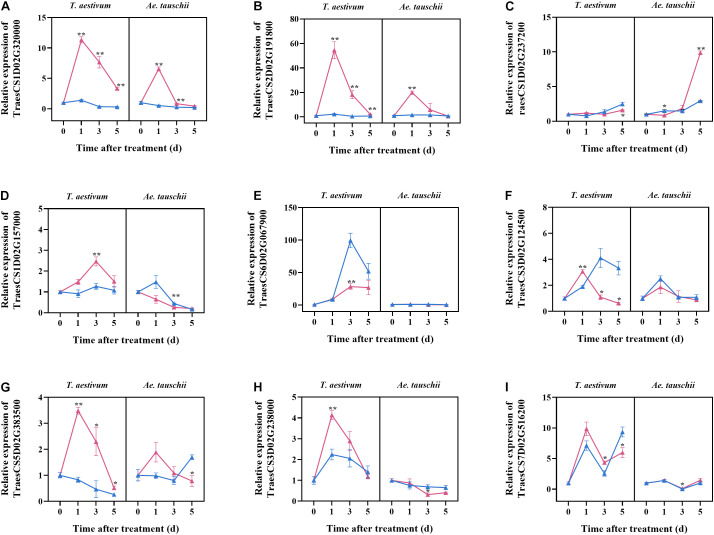
The relative expression levels of nine candidate genes selected from transcriptome analysis. The samples were collected from tillers buds of *T. aestivum* and *Ae. tauschii* at 0, 1, 3, and 5 days after 2,4-D isooctyl ester treatment. At 0 days after treatment, the relative expression level of target genes in untreated *T. aestivum* and *Ae. tauschii* seedlings was set 1, respectively. Red line represents seedlings with 2,4-D isooctyl ester treatment (treatment); blue line represents seedlings without 2,4-D isooctyl ester treatment (mock). Error bars represent the mean ± SD (*n* = 3). The significant difference between mock group and treatment group is indicated (*t*-test, *P < 0.05, **P < 0.01). The annotations of gene ID **(A–I)** are as shown in Table 2.

To validate the selected candidate genes responsible for the different tillering sensitivities of *T. aestivum* and *Ae. tauschii* to 2,4-D isooctyl ester, their expression at different times after treatment was assayed by qPCR. *T. aestivum* and *Ae. tauschii* showed similar dynamics of TraesCS1D02G320000 expression after 2,4-D isooctyl ester treatment; however, the degree of influence on gene expression in *T. aestivum* was clearly different from that in *Ae. tauschii*. For example, its expression level in *T. aestivum* after treatment was 11.34 times higher at 1 DAT than at 0 DAT but 6.55 times higher in *Ae. tauschii*. Similar results were also observed for TraesCS2D02G191800, TraesCS1D02G237200, TraesCS5D02G383500, and TraesCS7D02G516200. After 2,4-D isooctyl ester treatment, the expression levels of TraesCS1D02G157000 and TraesCS3D02G238000 increased at beginning and then decreased in *T. aestivum*, however, in *Ae. tauschii*, their expression levels gradually decreased with tiller bud elongation. For TraesCS6D02G067900 and TraesCS3D02G124500, treated *Ae. tauschii* displayed similar expression levels as mock groups at all time points, whereas expression levels were significantly different between the treatment and mock groups for at least one time point in *T. aestivum* (Figure 5). Collectively, the expressions of candidate genes responded differently to 2,4-D isooctyl ester in *T. aestivum* versus *Ae. tauschii*, confirming that the candidate genes may be involved in tillering differences.

### Changes in ABA and CTK Levels Differed Between the Two Species

**FIGURE 6:**
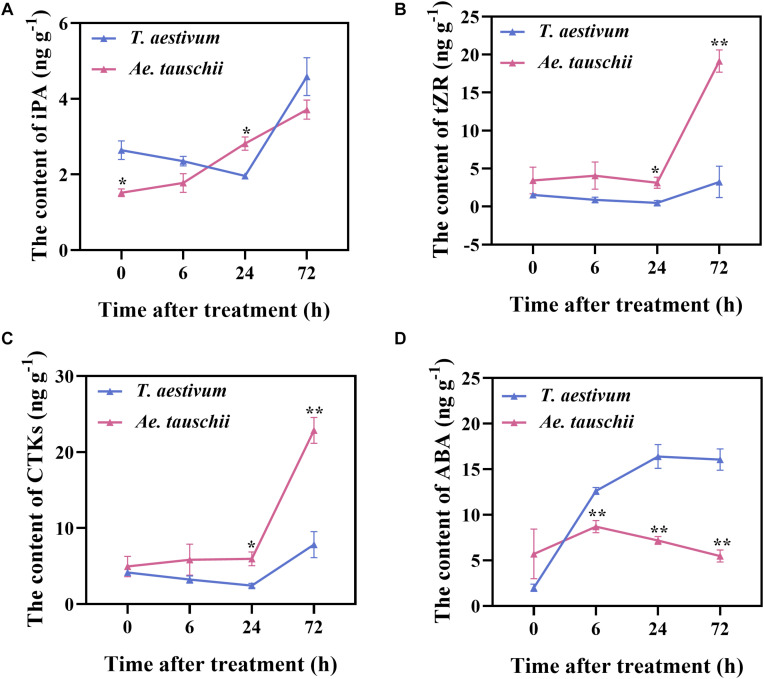
The levels of N6-isopentenyladenosine **(A)**, trans-zeatin-riboside **(B)**, total cytokinin **(C)**, and abscisic acid **(D)** detected in the 0.5 cm basal part of the stem in *T. aestivum* and *Ae. tauschii* at 0, 6, 24, and 72 h after 2,4-D isooctyl ester treatment. Error bars represent the mean ± SD (*n* = 3). The significant difference between *T. aestivum* and *Ae. tauschii* is indicated (*t*-test, *P < 0.05, **P < 0.01).

To further verify the RNA-seq results, the levels of IAA, CTK (iP, iPA, tZ, and tZR), ABA, GA_3_ and ACC in BPS were determined in *T. aestivum* and *Ae. tauschii* after 2,4-D isooctyl ester treatment. The concentrations of GA_3_, iP and tZ in BPS were distinctly lower than their limits of quantitation; thus, these endogenous hormones were not detected in either *T. aestivum* or *Ae. tauschii*. Total CTK (CTKs) levels in *Ae. tauschii* consistently increased after the application of 2,4-D isooctyl ester, whereas in *T. aestivum*, levels gradually decreased from 0 HAT to 24 HAT and clearly increased thereafter. A similar tendency was also observed for the single compounds iPA and tZR (Figures 6A–C). The iPA content in *T. aestivum* was significantly higher (*P* < 0.05) at 0 HAT than that in *Ae. tauschii* and then decreased to a lower level (*P* < 0.05) than that detected in *Ae. tauschii* at 24 HAT (Figure 6A). Compared with *T. aestivum*, tZR and CTKs levels in *Ae. tauschii* were significantly increased (*P* < 0.05) at 24 HAT and 72 HAT (Figures 6B,C). Collectively, 2,4-D isooctyl ester hampered CTK accumulation in *T. aestivum* compared with *Ae. tauschii*. The ABA concentration in *T. aestivum* sharply rose to a higher level (*P* < 0.01) than that in *Ae. tauschii* and then remained stable; however, in *Ae. tauschii*, the maximum level was observed at 6 HAT followed by a gradual decline with the outgrowth of tiller buds. After 2,4-D isooctyl ester treatment, *Ae. tauschii* exhibited clearly decreased ABA levels in BPS compared with *T. aestivum*, particularly at 72 HAT, when levels were 2.93-fold lower than those in *T. aestivum* (Figure 6D). This result indicates that 2,4-D isooctyl ester induced different responses in terms of ABA levels between *T. aestivum* and *Ae. tauschii*. For IAA and ACC, there was no observable difference in levels between the two plant species except at 6 HAT (*P* < 0.05) (Supplementary Figure 3). In addition, *T. aestivum* displayed roughly similar trends in ACC levels as *Ae. tauschii* following exposure to 2,4-D isooctyl ester (Supplementary Figure 3B). Clearly, ABA and CTK dominated the detected endogenous phytohormone response to 2,4-D isooctyl ester and exhibited different dynamics in levels between *T. aestivum* and *Ae. tauschii*.

### External ABA Inhibited the Outgrowth of Tiller Buds

**FIGURE 7:**
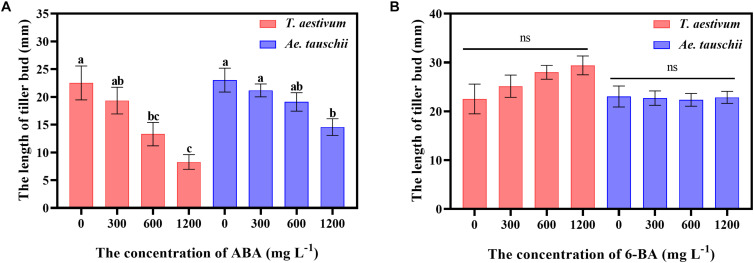
The effects of external spraying of abscisic acid **(A)** and 6-benzyl aminopurine **(B)** on tiller bud outgrowth in *T. aestivum* and *Ae. tauschii*. Error bars represent the mean ± SD (*n* = 3). Different letters represent significant differences (ANOVA, Student-Newman-Keuls test, *P* < 0.05, ns, no significant difference).

To clarify the precise roles of ABA and CTK in tiller bud growth, exogenous ABA and 6-BA were sprayed on *T. aestivum* and *Ae. tauschii*. External ABA significantly inhibited the outgrowth of tiller buds in *T. aestivum*, and the inhibitory effect was gradually enhanced with increasing ABA concentrations from 0 to 1200 mg L^–1^. A similar phenomenon was observed for *Ae. Tauschii* (Figure 7A). However, exogenously applied 6-BA had no significant effect on tiller bud growth in *T. aestivum* and *Ae. Tauschii* (Figure 7B).

## Discussion

An externally applied 2,4-D isooctyl ester spray negatively influenced tiller bud growth in *Ae. tauschii* and *T. aestivum*, and the inhibitory effects were enhanced with increasing 2,4-D isooctyl ester doses. It is well established that auxin participates in apical dominance and has a suppressive role in the tillering and branching of plants (Thimann and Skoog, 1934; McSteen, 2009), largely supporting our results. A similar phenomenon was observed for other plant species, such as rice and wheat (Liu et al.,2011a,b; Xu et al., 2015; Cai et al., 2018). Further analysis showed that *Ae. tauschii* tillering was more tolerant to 2,4-D isooctyl ester than *T. aestivum*. Cai et al. (2018) found that tiller bud growth in *T. aestivum* was completely inhibited by external application of IAA at a concentration of 60 mg L^–1^. However, we previously reported that the effect of 60 mg L^–1^ 2,4-D isooctyl ester on *Ae. tauschii* tiller bud elongation was very slight (Yu et al., 2020b), suggesting different tillering sensitivities to external auxin between the two plant species. This result is generally consistent with the present findings.

In the global analysis of the transcriptome, many more DEGs (22458) were detected in *T. aestivum* than in *Ae. tauschii* (291). A reason for this phenomenon may be that the *T. aestivum* genome was selected as a reference in transcriptome analysis and possesses ABD genomes that are generally larger than those in *Ae. tauschii* (Liu et al., 2012). This reason that the *T. aestivum* genome may not be suitable for *Ae. tauschii* was excluded because the proportion of the total sequences mapped to the reference genome was over 95%. *Ae. tauschii* and *T. aestivum* share the D genome, thus, this phenomenon may be due to that most DEGs in *T. aestivum* were from A and B genomes other than D genome. However, further analysis *T. aestivum* DEGs showed that the number of DEGs from different genomes were similar, approximately 6000 DEGs (Supplementary Figure 4). In addition, as shown in Figure 1C, obvious difference in growth phase of *T. aestivum* tiller buds between untreated and treated seedlings after 2,4-D isooctyl ester treatment may also be responsible for the above phenomenon. For example, *TB1*, *SPLs* and *D27s*, which are related with tiller bud development, exhibited different expression levels between untreated and treated *T. aestivum* seedlings, yet these genes were not differentially expressed in *Ae. tauschii*. In our study, *Ae. tauschii* tiller bud growth was also slightly inhibited by 2,4-D isooctyl ester to some extent, thus, there may be other reasons for this strange phenomenon, which needs to be studied further in the future. The mode of action of auxin herbicides interferes with several biochemical and physiological processes, including plant hormone events, ultimately leading to inhibited plant growth (Grossmann, 2010). As expected, transcriptome analysis of tiller buds indicated that the expression patterns of genes related to plant hormone metabolism and signal transduction responded differently to 2,4-D isooctyl ester in *Ae. tauschii* versus *T. aestivum*, which might lead to differences in the dynamics of endogenous hormone levels in the two plant species. Plant hormones implicated in the regulation of plant branching and tillering are well characterized (Smith et al., 2017; Wang et al., 2018). The different plant hormone responses induced by 2,4-D isooctyl ester likely result in different phenotypes of tillering in the two plant species. It has been proposed that external application of auxin regulates tiller and branch development by influencing gene expression in phytohormone biosynthesis and signaling, and endogenous hormone accumulation (Liu et al., 2011b; Xu et al., 2015), supporting the above speculation. Additionally, a total of nine candidate genes were identified from transcriptome analysis. qPCR validation showed that *T. aestivum* exhibited different dynamics in target gene expression levels from *Ae. tauschii*, confirming the candidate genes may participate in the different tillering sensitivities to 2,4-D isooctyl ester.

Among the detected plant hormones, the changes in ABA and CTK levels were quite different between *T. aestivum* and *Ae. tauschii*, demonstrating that ABA and CTK were most likely to be related to differences in tiller bud growth following exposure to 2,4-D isooctyl ester. However, no detectable levels of GA_3_, tZ or iP were observed in either *T. aestivum* or *Ae. tauschii*, suggesting that 2,4-D isooctyl ester had no obvious effect on these hormones or that they played roles in tillering at extremely low concentrations. Due to technological limitations in detecting these hormones in the present study, their precise roles in 2,4-D isooctyl ester-regulated tillering will need to be clarified in the future. Overproduction of ABA appears to be the crucial factor responsible for the actual phytotoxic reactions and inhibited growth in response to auxin herbicide (Grossmann, 2000, 2003). When 2,4-D isooctyl ester was sprayed on the two plant species, a large accumulation of ABA accompanied by severely inhibited tiller bud growth was observed in *T. aestivum*, while *Ae. tauschii* exhibited decreased ABA levels along with slightly restricted tiller bud elongation, indicating that there might be a synergetic relationship between ABA and 2,4-D isooctyl ester to restrain tillering. ABA has a stimulatory role in seed and bud dormancy (Shu et al., 2016; Nguyen and Emery, 2017), and recent evidence has revealed that it negatively influences tiller development in some plant species (Luo et al., 2019; Liu et al., 2020; Yu et al., 2020b), strongly supporting the above speculation. Moreover, in this research, external spraying of ABA significantly restricted tiller bud growth in *T. aestivum* as well as in *Ae. tauschii*, confirming its repressive regulation on tiller development. Several studies have reported that exogenous ABA application inhibited branching or tillering in some plant families, as was observed in *Arabidopsis* (González-Grandío et al., 2017), wheat (Cai et al., 2013) and rice (Liu et al., 2020), largely consistent with the present result.

In the present study, a 2,4-D isooctyl ester spray markedly contributed to the production of CTK in both *T. aestivum* and *Ae. tauschii*; however, the stimulatory effect was larger in *Ae. tauschii* compared with that in *T. aestivum*. Participation of CTK in the release of apical dominance and the promotion of tiller bud elongation has been well established (Galuszka et al., 2008; Shimizu-Sato et al., 2009). In wheat, a significant positive relationship was found between the levels of endogenous zeatin and tiller bud growth (Cai et al., 2018). CTK played an activating role in plant density-related regulation of tiller development in *Ae. tauschii* (Yu et al., 2020b). Consistently, overproduction of CTK was detected in *Ae. tauschii* after 2,4-D isooctyl ester treatment, while tiller bud growth was slightly inhibited; however, a large decrease in tiller bud elongation accompanied by less accumulation of CTK was observed in *T. aestivum*. Therefore, CTK was positively associated with tiller bud growth in present study. However, whether CTK is involved in the 2,4-D isooctyl ester-regulated tillering of *T. aestivum* and *Ae. tauschii* needs to be studied further in the future. Due to the positive role of CTK in tiller bud development, the obvious growth of *Ae. tauschii* tiller bud when exposed to 2,4-D isooctyl ester would require an accumulation of CTK, which might lead to a great elevation of CTK contents in shoot base of *Ae. tauschii*. Based on that, changes of CTK may be due to the difference in tiller bud status. Nevertheless, auxin plays an indirect role in the modulation of tiller development because it cannot enter the tiller bud (Booker et al., 2003). It has been proposed that CTK is the second messenger that transmits auxin signals to tiller buds (Tanaka et al., 2006; Shimizu-Sato et al., 2009). Additionally, Liu et al. (2011b) found that exogenous auxin arrested the development of tiller buds in rice by negatively regulating *OsIPT* expression in nodes, which encodes a crucial enzyme involved in CTK biosynthesis, supporting that 2,4-D isooctyl ester may regulate tillering in *T. aestivum* and *Ae. tauschii* through disturbing CTK metabolism. Interestingly, there was no distinct effect of external 6-BA application on tiller bud growth. Similar to our observation, the tillering of *Miscanthus × giganteus* was not influenced by exogenously applied thidiazuron, a cytokinin-like compound (Schmidt et al., 2017). Yu et al. (2020b) also found that external spraying of 6-BA at concentrations of 60 mg L^–1^ and 120 mg L^–1^ had no significant effect on *Ae. tauschii* tiller bud growth. The reason for the result may be that externally applied CTK or cytokinin-like compounds cannot influence the levels of endogenous CTK, given the finding that exogenous zeatin (Z) spray increased Z levels for a short period, after which there was no significant difference between control and Z treatment groups (Cai et al., 2018).

## Conclusion

In conclusion, the inhibitory effect of 2,4-D isooctyl ester on *T. aestivum* tiller bud growth was more pronounced than that in *Ae. tauschii*. A total of nine candidate genes implicated in the different tillering responses to 2,4-D isooctyl ester were selected from transcriptome analysis and validated by qPCR. The auxin biosynthesis and signaling in *T. aestivum* and *Ae. tauschii* responded differently to this herbicide, which may be responsible for the varied tillering. Furthermore, ABA and CK were most likely to be related to 2,4-D isooctyl ester-regulated tillering of *T. aestivum* and *Ae. tauschii*, and differential changes induced by the herbicide in the two plant species might result in their different tillering sensitivities. The results obtained from this work will help to further elucidate the mechanism underlying the regulation of tillering by auxin and might provide new insight into the inhibition of tillering in *Ae. tauschii* by auxin herbicides while avoiding side effects on *T. aestivum*.

## Data Availability Statement

The raw sequence data has been deposited in the NCBI Sequence Read Archive (SRA) database with the accession number of PRJNA680816.

## Author Contributions

XL conceived the study and provided writing assistances. HY conducted the experiments and participated in the manuscript writing. PC, MJ, and SH provided assistances in experiment conduction. HC participated in the seed collection. JC provided writing assistances. All authors contributed to the article and approved the submitted version.

## Conflict of Interest

The authors declare that the research was conducted in the absence of any commercial or financial relationships that could be construed as a potential conflict of interest.
