# Supplementary material for: Regulation of 2,4-D Isooctyl Ester on Triticum aestivum and Aegilops tauschii Tillering and Endogenous Phytohormonal Responses
Source: Front Plant Sci. 2021 Apr 28;12:642701. doi: 10.3389/fpls.2021.642701 (PMC8113871; doi:10.3389/fpls.2021.642701)
Supplement: Supplementary file 1 [file Data_Sheet_1.docx]

Supplementary Material


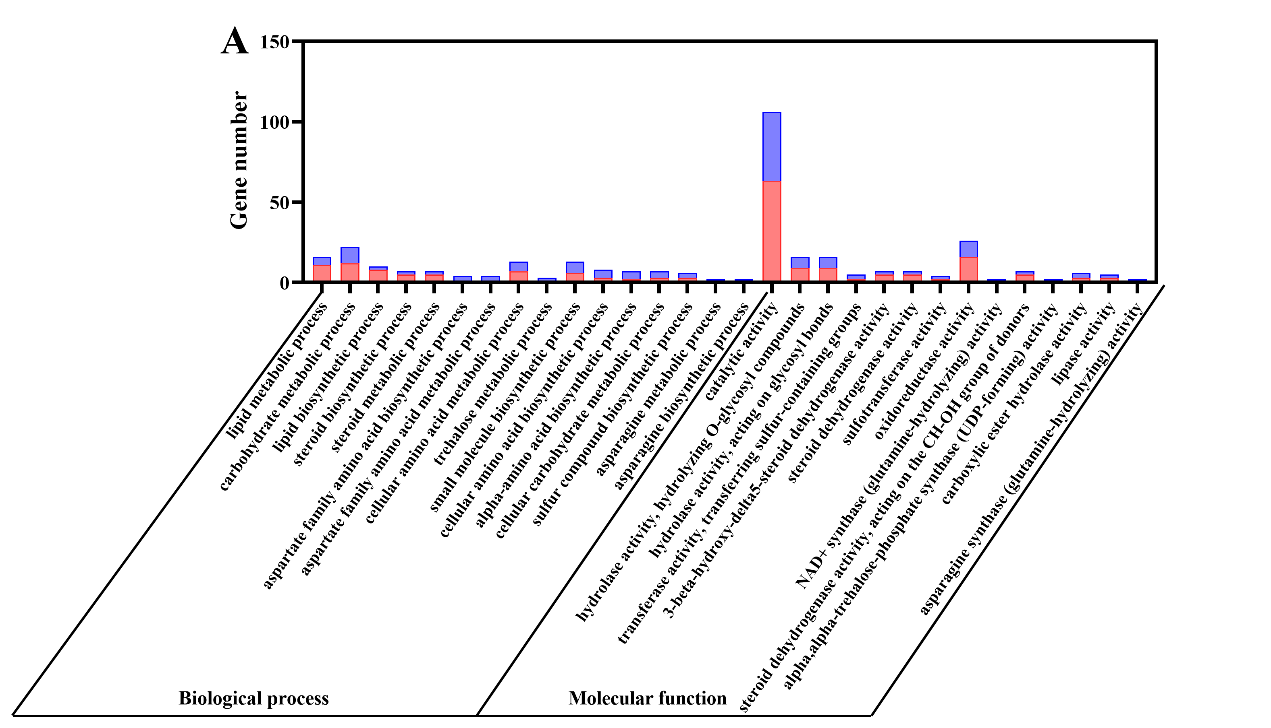

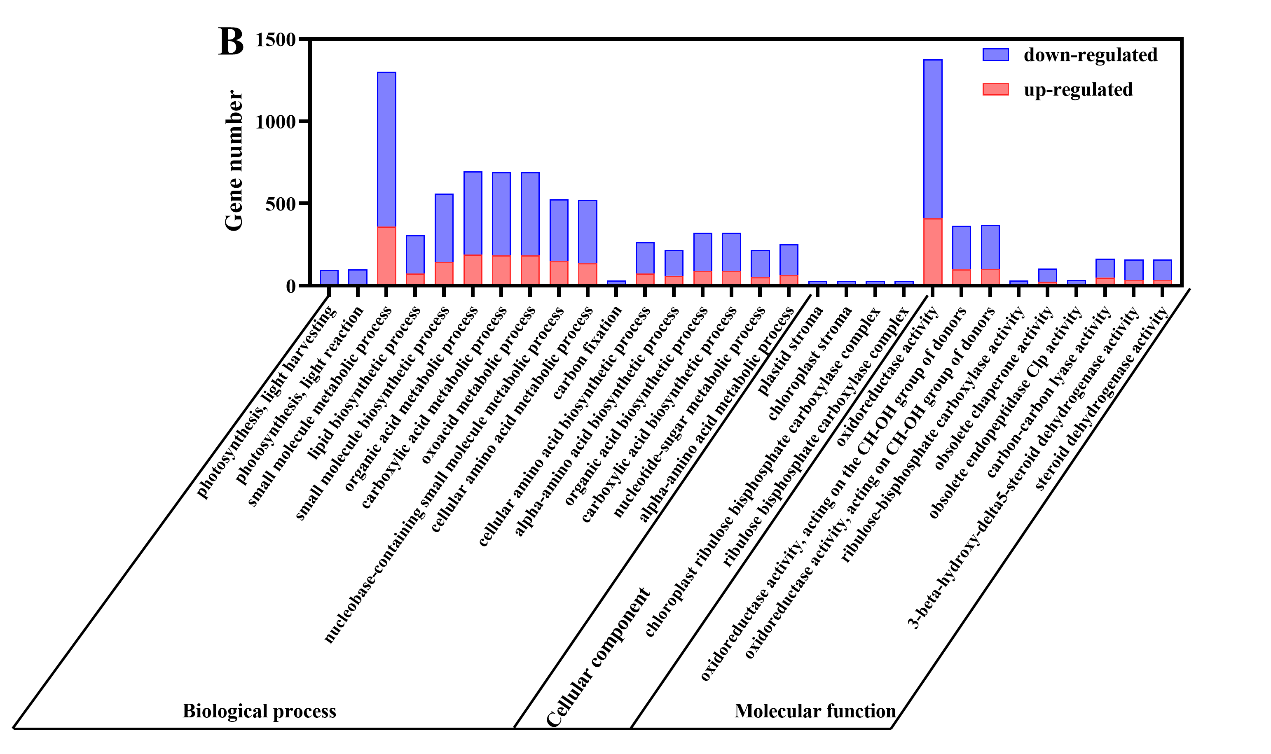


**Supplementary Figure 1.** GO enrichment analysis of differentially expressed genes (DEGs) in *Aegilops tauschii* (A) and *Triticum aestivum* (B).


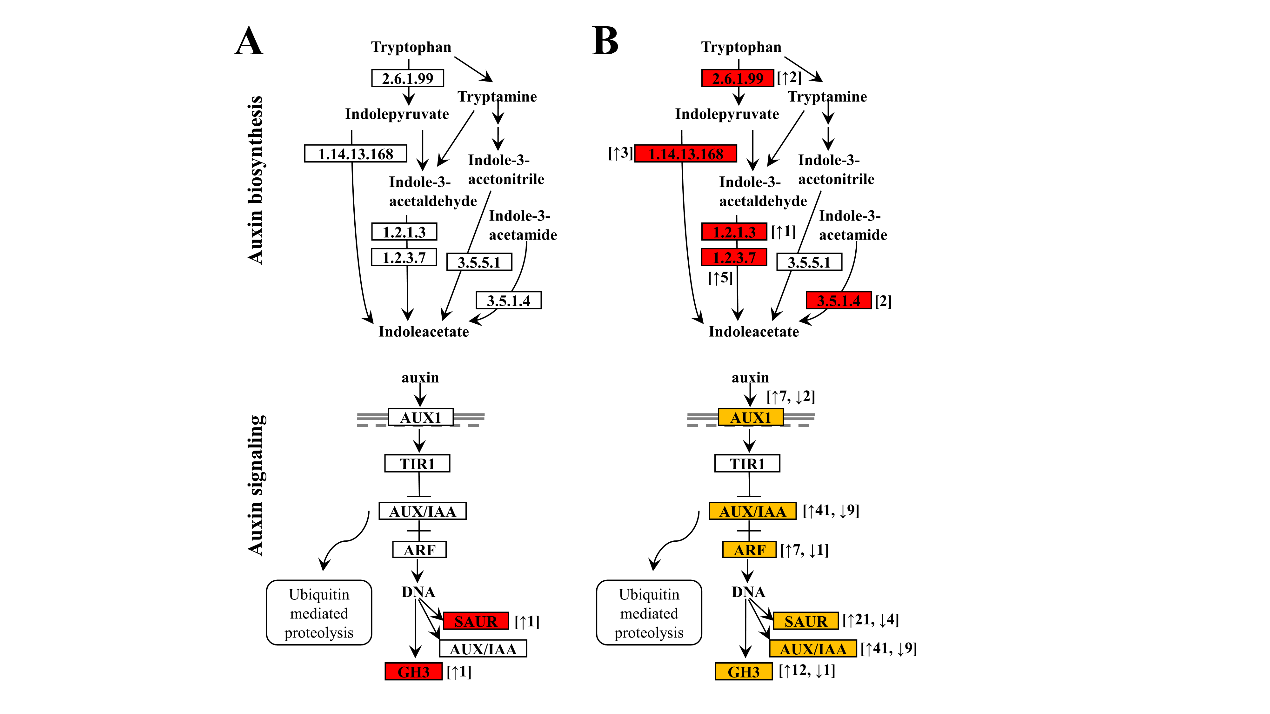

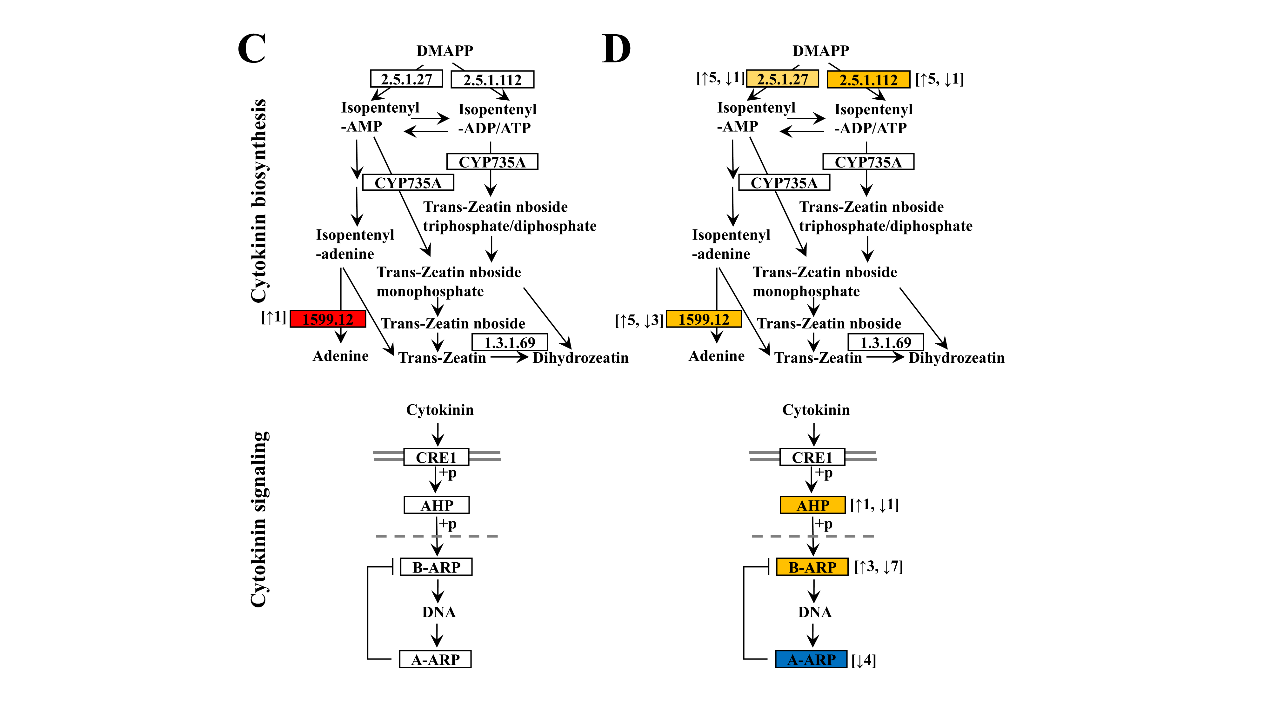


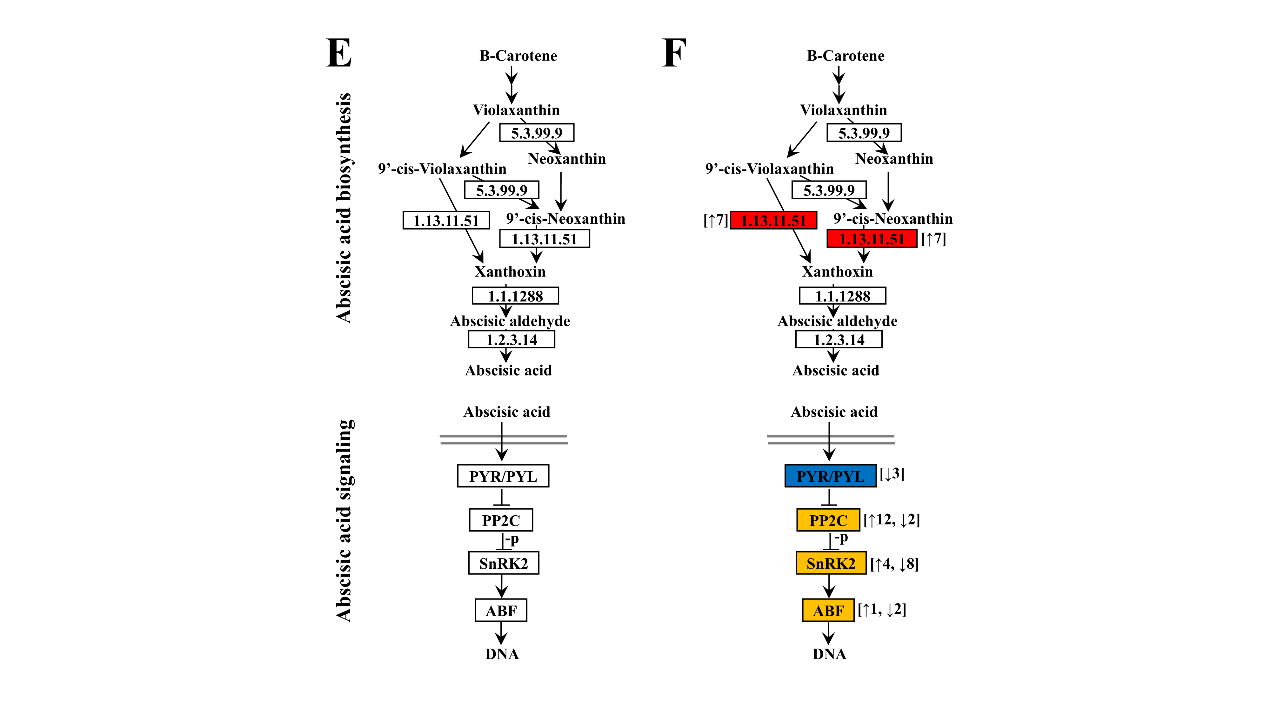

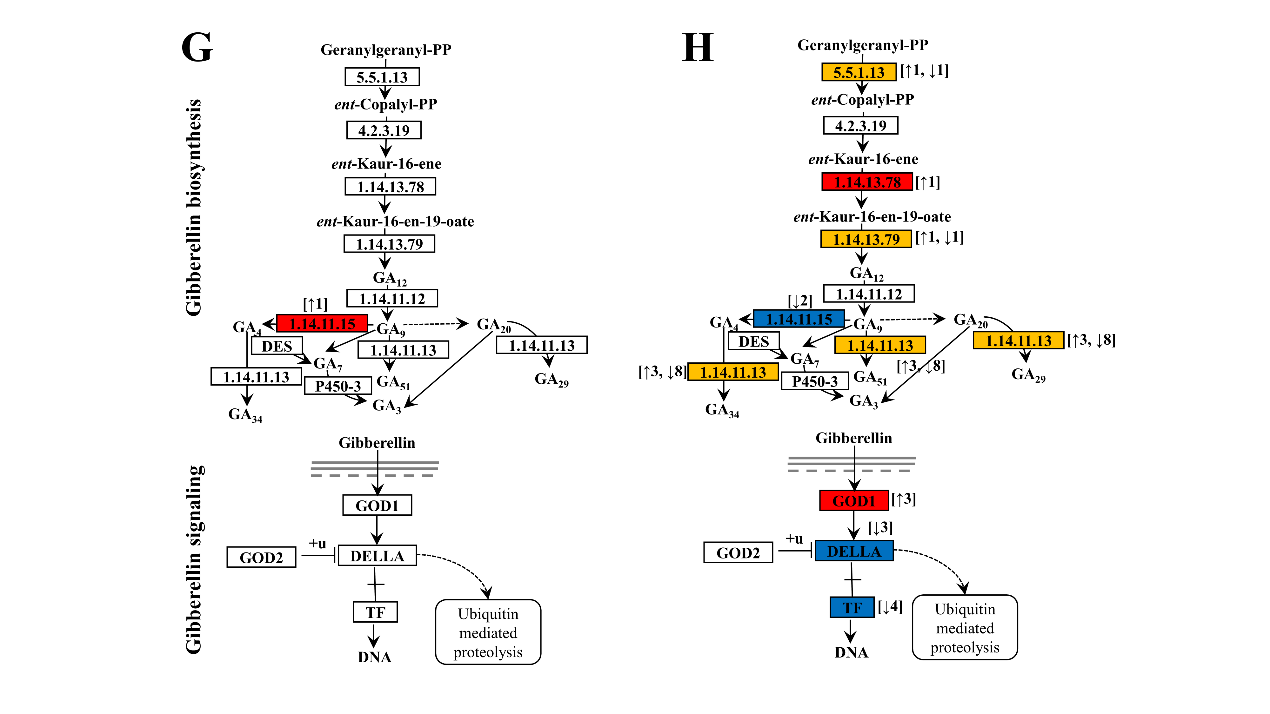


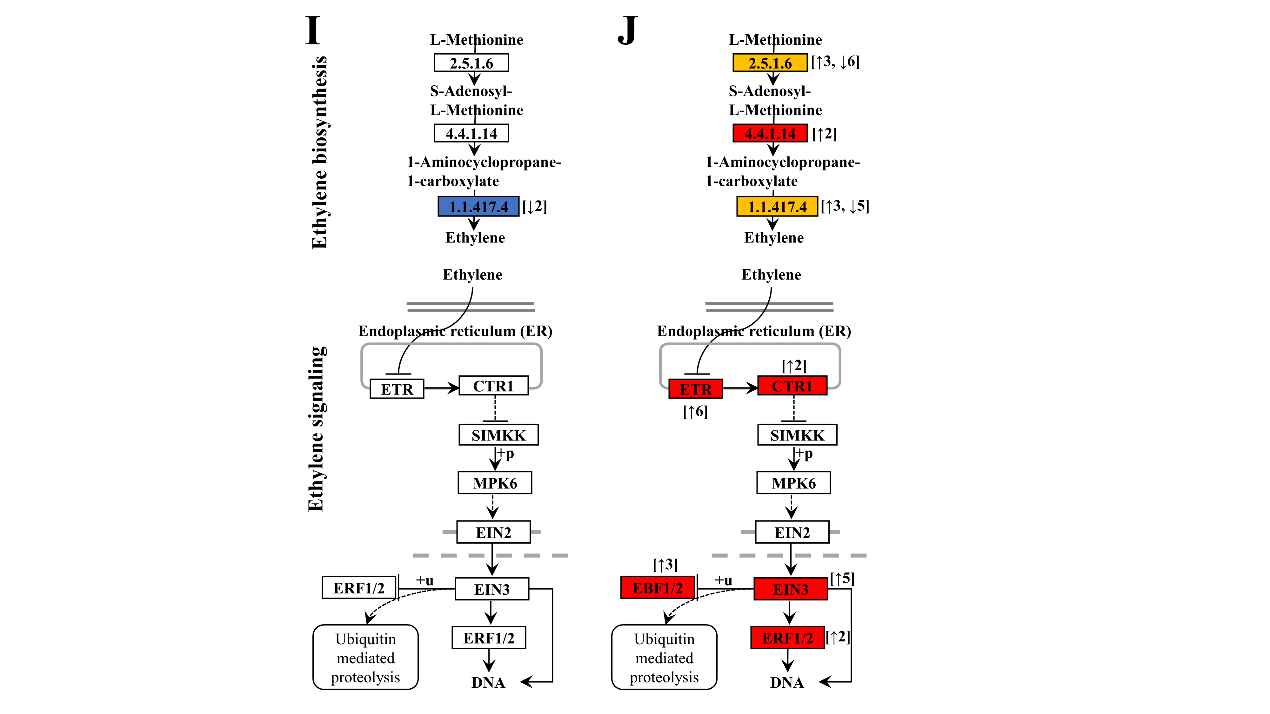


**Supplementary Figure 2.** Plant hormone biosynthesis and signal transduction pathways in *Ae. tauschii* (A, C, E, G and I) and *T. aestivum* (B, D, F, H and J). Genes in the red and blue boxes are upregulated DEGs and downregulated DEGs, respectively. Genes in the yellow boxes include upregulated DEGs and downregulated DEGs. The figures in square brackets represent the number of DEGs. ↑, upregulated DEGs; ↓, downregulated DEGs.


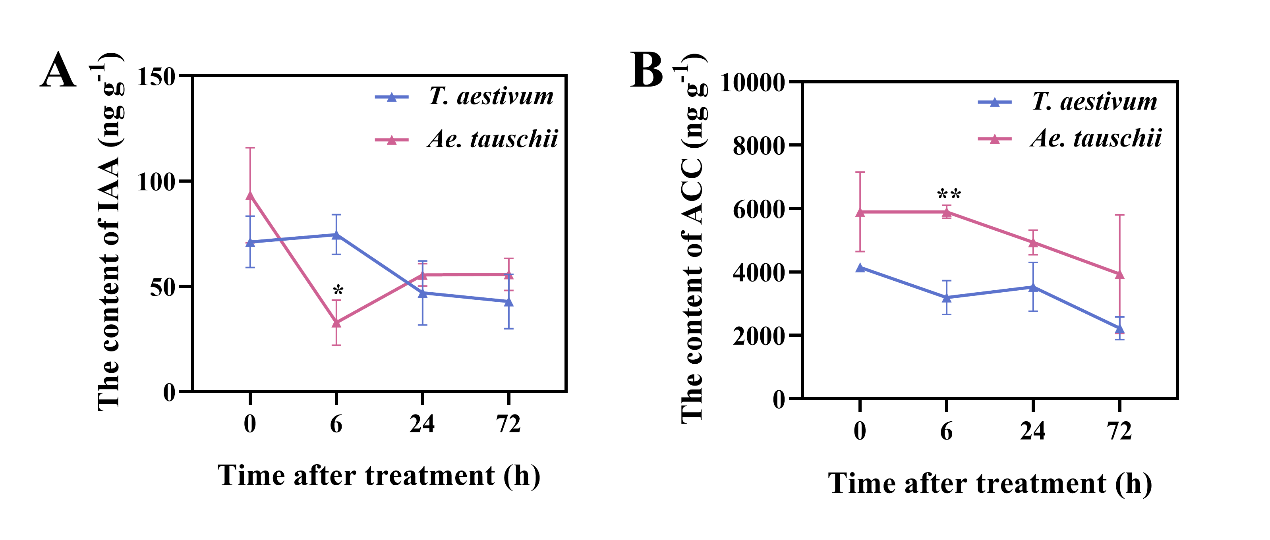


**Supplementary Figure 3.** The levels of indole-3-acetic acid (A) and 1-aminocyclopropanecarboxylic acid (B) detected in the 0.5 cm basal part of the stem in *T. aestivum* and *Ae. tauschii* at 0, 6, 24 and 72 hours after 2,4-D isooctyl ester treatment. Error bars represent the mean ± SD (n=3). The significant difference between *T. aestivum* and *Ae. tauschii* is indicated (t-test, *, *P* < 0.05, **, *P* < 0.01).


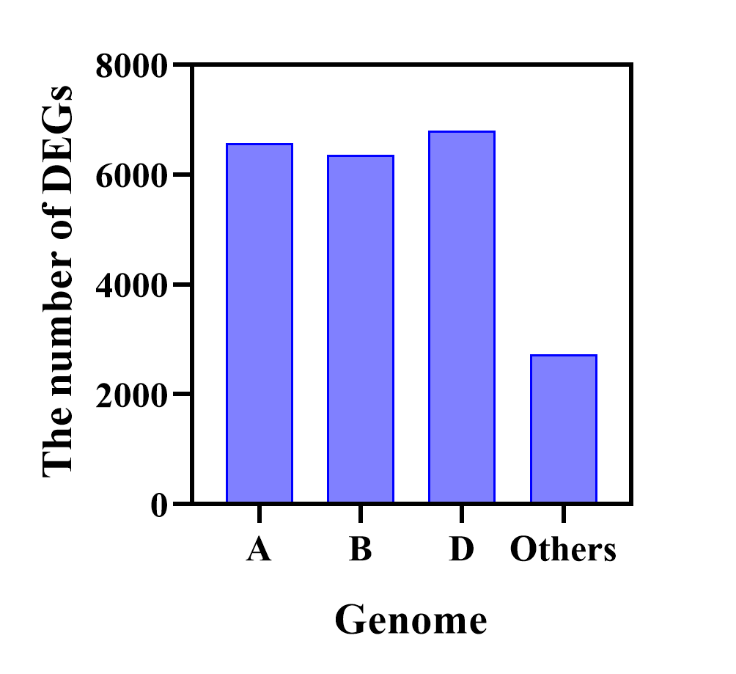


**Supplementary Figure 4.** The number of *T. aestivum* DEGs from different genomes. Others represent novel genes and their locations on genome are unclear.

**Supplementary Table 1.** Sequences of primers used in the study.

| Gene ID | Forward primer (5'-3') | Reverse primer (5'-3') |
| --- | --- | --- |
| TraesCS1D02G320000 | GCCCAACACCAAGTACATAGACG | CCGCCGCCGTAGTAGTCG |
| TraesCS2D02G191800 | CATCGGGACTCTCAAGTACTAC | GAACTCGAAGTACCCCATGTTC |
| TraesCS1D02G237200 | GCAATGTGAACGAACTGGAGAT | ATTGACCTAGACCACCAAGAGC |
| TraesCS1D02G157000 | TGAGCCACATAGCGTCCCACC | CTCATCTCCTCCATCCTCACCC |
| TraesCS6D02G067900 | TGAACCAGCCCAACAGCCTC | TGACGCACAAAACCACCCACC |
| TraesCS3D02G124500 | CGCCCATCTCCTCCTTCTTCTC | GCAGAAGTGGCGGTAGTCGT |
| TraesCS5D02G383500 | CCAGCCCACCATGATCCACG | CCACCCACACCATCTCCGAC |
| TraesCS3D02G238000 | ACCACAAGCCAGACCGAC | ACACCACATCCCACAGGC |
| TraesCS7D02G516200 | GGACTGGGGCTTCTTCTGGGTG | AGATGCTCGTCGTAGTGGGCGTA |
| *Tubulin* | CCGTTACCTCACCGCCTCT | ATCCACTCCACAAAGTAGGACG |

**Supplementary Table 2.** Multiple reaction monitoring mode parameters of the target compounds.

| Compound | Molecular formula | ESI mode | Precursor ion (m/z) | Quantitative ion (m/z) | Qualitative ion (m/z) |
| --- | --- | --- | --- | --- | --- |
| (+)-Abscisic acid | C_15_H_20_O_4_ | ESI^-^ | 263.0 | 153.0 | 219.0 |
| trans-zeatin | C_10_H_13_N_5_O | ESI^+^ | 220.0 | 136.0 | 202.0 |
| trans-zeatin-riboside | C_15_H_21_N_5_O_5_ | ESI^+^ | 352.0 | 220.0 | 136.0 |
| N6-(delta 2-Isopentenyl)-adenine | C_10_H_13_N_5_ | ESI^+^ | 204.0 | 136.0 | 148.0 |
| N6-isopentenyladenosine | C_15_H_21_N_5_O_4_ | ESI^+^ | 336.0 | 204.0 | 136.0 |
| Indole-3-acetic acid | C_10_H_9_NO_2_ | ESI^+^ | 176.0 | 130.0 | 103.0 |
| Gibberellin A3 | C_19_H_22_O_6_ | ESI^-^ | 345.0 | 143.0 | 239.0 |
| 1-aminocyclopropanecarboxylic acid | C_4_H_7_NO_2_ | ESI^+^ | 102.0 | 56.0 | 56.0 |
